# Bioprinted Bladder Cancer Organoids Model System for Prediction of Chemotherapy Response and Drug Screening

**DOI:** 10.3390/ijms27136082

**Published:** 2026-07-07

**Authors:** Randall G. Bissette, Zachary Congress, Gemma Nomdedeu-Sancho, Nadeem Wajih, Krishnaiah Maddeboina, Shay Soker

**Affiliations:** 1 Wake Forest Institute for Regenerative Medicine, Wake Forest University School of Medicine, Winston-Salem, NC 27101, USA; 2 Department of Urology, Wake Forest University School of Medicine, Winston-Salem, NC 27157, USA; 3 Atrium Health Levine Cancer Center, Charlotte, NC 28204, USA

**Keywords:** organoids, bladder cancer, small molecule inhibitor, drug testing, CDK inhibitor

## Abstract

Bladder cancer is the fifth most common cancer in the United States, causing approximately 17,000 deaths annually. Due to its vast genetic and molecular heterogeneity, presentation, prognosis, and therapeutic response vary greatly between individuals. To improve patient outcomes, there is a need for better drug-screening platforms. The genetic heterogeneity of bladder cancer often leads to chemotherapy resistance or low response rates. Moreover, chemotherapies are often contraindicated in patients with select comorbidities. Organoids offer a better option to replicate the tumor microenvironment than traditional 2D cell cultures, improving drug development and personalized therapy. In this study, we bioprinted gelatin-methacrylol (GelMA)-based organoids containing bladder cancer cell lines of different grades to model muscle-invasive bladder cancer. In the organoids, we observed distinct grade-dependent tumor proliferation and progression dynamics. Treatment with standard-of-care chemotherapies revealed a grade-dependent tumor response consistent with in vivo patient data, highlighting the suitability of these organoids for rapid, reliable drug testing. Lastly, we used the organoids to test LCI139, a novel small-molecule inhibitor of PI3K, CDK4/6, and CDK9 designed for the treatment of epithelial cancers, underscoring the potential of our model to evaluate the efficacy of newly developed drugs. The ability to quickly biofabricate reproducible bladder cancer organoids that are adaptable to different tumor grades represents a novel strategy to create an in vitro platform with strong potential to predict treatment outcomes of bladder cancer patients.

## 1. Introduction

In 2025, there were an estimated 84,870 new bladder cancer cases, representing 4.2% of all new cancer diagnoses. Bladder cancer was responsible for 17,420 deaths, and 2.8% of cancer-related mortality [1]. On presentation, 70–75% have non-muscle-invasive disease, and 5% have metastasis. However, over a 5-year period, 40–80% of non-muscle-invasive cases progress to muscle-invasive bladder cancer (MIBC) [2]. The 5-year survival rate of MIBC is estimated to be 60–70%, with this number cratering to 5% when distant metastasis is present [3].

Treatment of bladder cancer depends on risk stratification, which considers clinical staging and pathological grading, with more aggressive approaches reserved for patients at higher risk. Staging is defined by tumor invasion depth, and grading is based upon the morphological appearance of tumor cells on pathology. Muscle invasion is considered a crucial factor in determining appropriate treatment and rapidly elevates the severity of intervention. For low- or intermediate-risk bladder cancer, intravesical chemotherapy—commonly gemcitabine—should be performed at the time of transurethral resection of the bladder tumor (TURBT) [4]. For non-metastatic muscle-invasive bladder cancer, neoadjuvant cisplatin chemotherapy should be offered prior to cystectomy in surgically eligible patients. Bladder-sparing approaches may also be considered with radiation-sensitizing chemotherapy as a cornerstone [5]. Once metastasis occurs, treatment may expand to adjuvant radiation and combination therapy with immunotherapy in addition to chemotherapy [6]. A pivotal randomized clinical trial showed that less than 40% of patients responded to neoadjuvant chemotherapy when combined with radical cystectomy [7]. Critically, management algorithms vary from patient to patient, and treatment is individualized, necessitating personalized medical recommendations in these circumstances.

Bladder cancer is heterogeneous both molecularly and pathologically, further emphasizing the need for tailored and targeted therapies [8,9]. Muscle-invasive bladder cancer is characterized by a high genetic mutation rate [10]. Variable genetic expression influences tumor aggressiveness and clinical outcomes [11]. Bladder cancer treatment resistance may be influenced by genetic alterations leading to disease progression [12,13]. For example, cisplatin-resistant tumors have demonstrated selection for subclonal mutations following chemotherapeutic treatment. Furthermore, intratumoral heterogeneity predicts worse overall survival in those cisplatin-resistant individuals [14].

With the prevalence, mortality, and challenging management pathway surrounding bladder cancer, better tools for drug screening are needed. Bladder cancer organoids have been minimally but increasingly used as translational models to explore individualized and targeted therapies. Not only do they recapitulate the tumor microenvironment (TME) and tumorigenesis, but they can also capture the tumor’s unique genetic, phenotypic, and histopathological features, enabling the evaluation of response to potential therapies [15,16,17]. Although diverse fabrication methods for bladder cancer organoids exist, few have described the use of Gelatin-methacrylol (GelMA) hydrogel structure. GelMA, as a biomaterial, has become increasingly relevant for bioprinting applications due to its UV-induced cross-linking capabilities and physicochemical tunability, which allow precise control of the mechanical properties of the resulting organoid microenvironment [18]. Also, because it is a biocompatible, porous hydrogel, it enables extracellular adhesion through RGD motifs and supports cell proliferation and migration [19].

Here, we describe our methodology for bioprinting GelMA-based bladder cancer organoids. We report differences in organoid cellular growth characteristics and drug treatment responses across distinct grades of bladder cancer. We also evaluate the use of a novel in silico-designed small-molecule CDK inhibitor, LCI139, in bioprinted bladder cancer organoids, which demonstrated the potential of this new epithelial-targeting agent for MIBC treatment. Our results highlight the utility of bladder cancer organoids as screening tools that capture the unique characteristics of different tumor profiles and aggressiveness, and their potential to aid pre-clinical drug development.

## 2. Results

### 2.1. GelMA-Based Bioprinted Bladder Cancer Organoids (BCaOs) from Different-Grade Bladder Cancer Cell Lines

**Figure 1:**
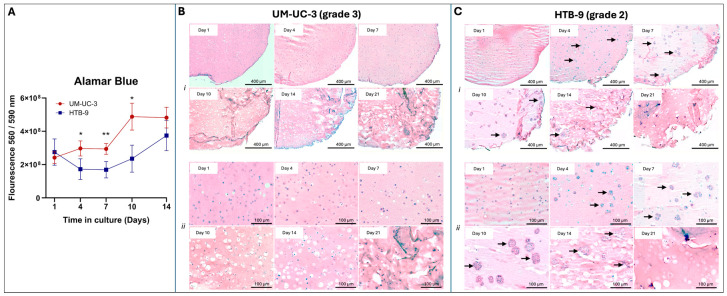
Viability and cellular organization of GelMA-based bladder cancer organoids differ based on tumor grade. (**A**). Progressive increase in cellular viability within organoids. (*n* = 5 BCaOs per condition. Error bars represent standard deviation. Statistical significance was determined using a two-tailed Student’s *t*-Test. * *p* < 0.05, ** *p* < 0.01). (**B**). H&E staining of paraffin-embedded UM-UC-3 organoid sections (grade 3). (**i**). 10× magnification. (**ii**). 40× magnification. (**C**). H&E staining of paraffin-embedded HTB-9 organoid sections (grade 2). Arrows indicate examples of cell clusters on days 4–14. (**i**). 10× magnification. (**ii**). 40× magnification.

Bladder cancer organoids were established using HTB-9 (grade 2) and UM-UC-3 (grade 3) cell lines, both of which are muscle-invasive. Cells were resuspended in an 8% GelMA-based hydrogel (bioink) at a density of 10^6^ cells/mL, and 10 µL droplets were crosslinked via UV-light exposure using lithium phenyl-2,4,6-trimethylbenzoylphosphinate (LAP) as a crosslinker. Alamar Blue assay of cellular viability for grade 3 bladder cancer organoids showed generally stable viability for days 1 to 7, followed by an increase in viability at days 10 to 14 (Figure 1A). Grade 2 bladder cancer organoids demonstrated a decrease in viability initially, followed by an increase in viability around days 7 to 14. These results suggest that the microenvironment within these organoids supports cell growth and proliferation of higher-tumor-grade cells, while better representing in vivo tumor characteristics in a 3D environment compared to 2D models.

Differing grades of bladder cancer cell lines exhibited distinct behavior in our organoid constructs. Throughout the culture period, grade 3 bladder cancer cell organoids demonstrated homogeneous cell dispersion as observed by H&E staining (Figure 1B). In contrast, grade 2 bladder cancer cells formed cellular clusters, arising around day 4, that increased in size throughout the 3-week culture period. (Figure 1C). These clusters were accompanied by decreasing cell density within the organoids. The GelMA-based structure of the organoids from both cell lines exhibited notable degradation by day 21, which substantially affected the maintenance of organoids in culture for long-term analysis. Collectively, organoids from higher-grade cancer cells demonstrated better survival, consistent with more aggressive tumors. Lower grades, conversely, needed cell-adhesion into clusters for survival, consistent with lower aggressiveness [20].

### 2.2. BcaOs Show Grade-Dependent Proliferation Dynamics

To assess the growth and proliferation patterns of bladder cancer cells from different grades within organoid constructs, Ki67 IHC staining was performed on paraffin-embedded organoid cross sections. IHC with EpCAM was also used to identify the epithelial cell borders.

**Figure 2:**
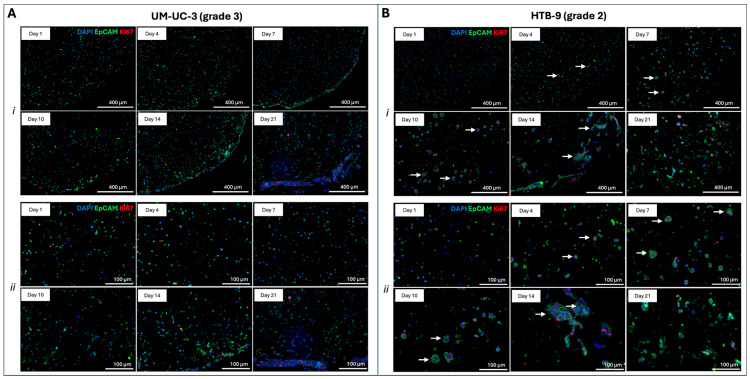
Cellular proliferation patterns within organoids. (**A**). Ki67 and EpCAM IHC staining of paraffin-embedded UM-UC-3 (grade 3) organoids’ cross sections. (**i**). 10× magnification. (**ii**). 40× magnification. (**B**). Ki67 and EpCAM IHC staining of paraffin-embedded HTB-9 (grade 2) organoid cross sections. Arrows indicate examples of cell clusters with Ki67 markers on days 4–14. ((**i**). 10× magnification. (**ii**). 40× magnification.) (DAPI (blue), EpCAM (green), and Ki67 (red)).

Grade 3 bladder cancer cell lines demonstrated a consistent Ki67 pattern over 14 days with minimal but evenly distributed staining throughout (Figure 2A). On day 21 of culture, proliferation was observed in aggregates at the borders of the organoid, possibly indicating enhanced tumor proliferation in areas of higher surface tension. Over the culture period, EpCAM staining in these grade 3 organoids became more diffuse and less membrane-localized, suggesting a loss of epithelial markers towards a mesenchymal, invasive phenotype.

Grade 2 bladder cells, on the other hand, showed proliferation within distinct cell clusters (Figure 2B), with proliferation markers increasing from day 4 to 14 days of culture. By this point, larger clusters of cells were notable for Ki67 staining throughout. Despite a reduction in average cluster size on day 21, cells within these clusters still retained a noticeable level of proliferation.

Overall, in grade 2 cells, proliferation was observed in these clusters but not in single cells outside the clusters, whereas grade 3 cells were spreading more throughout the organoids. These findings here are consistent with known cancer cell behavior, where cells from more aggressive tumor grades are typically more pro-migratory [20].

### 2.3. BcaOs Show Grade-Dependent Response to Standard-of-Care Chemotherapy Agents

**Figure 3:**
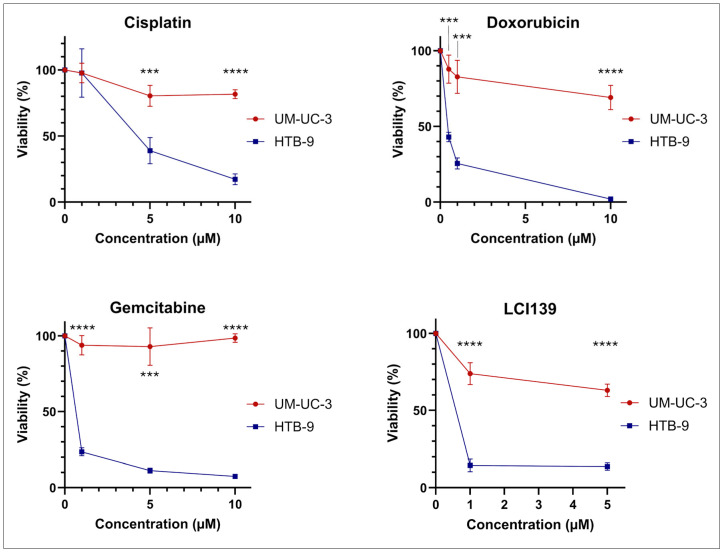
BCaOs response to chemotherapeutic agents. Cellular viability in organoids from each cell line after 72 h of exposure to various chemotherapeutic agents. (*n* = 5 BCaOs per condition. Error bars represent standard deviation. Statistical significance was determined using a two-tailed Student’s *t*-Test. *** *p* < 0.001, **** *p* < 0.0001).

To assess BCaO’s capability to serve as a platform for drug screening, organoids from each cell line were treated with various common chemotherapeutic agents. We included agents of multiple different classifications, including Cisplatin (alkylating agent), Doxorubicin (anthracycline), and Gemcitabine (antimetabolite). These treatments were applied on day 2 of cell culture, and cell viability was determined 72 h later, on day 5. For all chemotherapeutic agents used, there was significantly greater drug response in the grade 2 bladder cancer cell line compared to grade 3 (Figure 3). Chemotherapies revealed a grade-dependent tumor response (*n* = 5) with grade 2 versus grade 3 viability reduction as follows: Cisplatin, 61% versus 20% at 5 µM (*p* < 0.001), 83% versus 18% at 10 µM (*p* < 0.0001); Doxorubicin, 57% versus 12% at 0.5 µM (*p* < 0.001), 74% versus 17% at 1 µM (*p* < 0.001), 98% versus 31% at 10 µM (*p* < 0.0001); Gemcitabine, 76% versus 6% at 1 µM (*p* < 0.0001), 89% versus 7% at 5 µM (*p* < 0.001), 93% versus 1% at 10 µM (*p* < 0.0001).

To determine our model’s utility as a drug development platform, we also tested LCI139, a novel small-molecule inhibitor not yet used clinically. LCI139 is a small-molecule CDK9/CDK4/6/PI3K triple inhibitor whose chemical properties were optimized to increase PI3K p110α inhibition potency to better target cancers derived from epithelial cells [21]. LCI139 has shown effectiveness in endometrial carcinoma as well as *MYC*-driven mantle cell lymphoma [22], but it has not been previously tested for muscle-invasive bladder cancer. Bladder cancer tissue demonstrates higher CDK9 expression than normal urothelium [23], and alterations in the CDK4/6 pathway are commonly observed in these tumors [24]. The PI3K pathway has been shown to be activated in over 40% of bladder urothelial cancers and has been proposed as a potential therapeutic target [25], therefore making bladder cancer a good candidate for LCI139. Consistent with findings with common chemotherapeutics, LCI139 treatment resulted in a significantly greater drug response in grade 2 organoids than in grade 3 across all drug doses. Viability reduction in grade 2 versus grade 3 BCaO was as follows: 85% versus 26% at 1 µM (*p* < 0.0001), 86% versus 37% at 5 µM (*p* < 0.0001). Aside from the grade-dependent response, the LCI139 treatment was also notable in that the response to this drug by the grade 3 organoids was greater than that of any of the other drugs tested.

**Figure 4:**
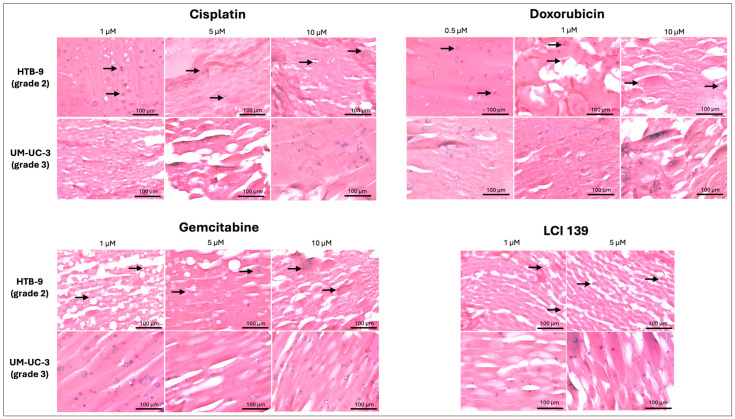
H&E staining of organoids after chemotherapy treatment shows grade-dependent differences in spatial tissue architecture. Arrows indicate loss of cell cluster formation in grade 2 organoids.

Post-chemotherapy changes in tissue architecture were distinct and grade-dependent between BCaOs of grade 2 and 3 lines. Specifically, chemo-resistant grade 3 organoids preserved their cellular distribution, displaying remaining viable individual cells similar to the controls (Figure 4). Conversely, chemo-sensitive grade 2 constructs exhibited a marked reduction in cell density and a widespread disruption of the epithelial clusters observed in the untreated controls, thus capturing the morphological and cell behavior changes derived from treatment.

Overall, these organoids allow us to discern grade-based responses in vitro, making them a suitable model for therapy testing and drug development.

## 3. Discussion

Muscle-invasive bladder cancer remains a clinical challenge due to its high molecular and clinical heterogeneity [8,9]. High genetic rates modify tumor aggressiveness and response to therapy [12,13], making it difficult to establish standardized treatment regimens that work for all patients. Treatment for muscle-invasive bladder cancer usually involves the use of cisplatin as a first-line chemotherapeutic agent [5]. Nonetheless, cisplatin is not indicated for all patients; it cannot be administered to patients with poor renal function, hearing loss, and peripheral neuropathy, among other co-morbidities [26]. In addition, a proportion of patients develop resistance to cisplatin treatment over time. In these cases, therapeutic choice relies on standard chemotherapy algorithms that cannot predict success beforehand, often subjecting patients to unnecessary toxicity from ineffective treatments. There is a need for both novel therapeutic options with fewer contraindications and reliable human preclinical platforms to test them.

Organoids offer a great opportunity to overcome these hurdles. Recently, bladder cancer organoids developed from both cell lines and patient-derived primary cancer cells have been used to model tumor development, genetic evolution, and drug response to chemo- and immunotherapies [15,27,28,29]. However, most of these models have relied on Matrigel as the ECM component. Due to its undefined composition, Matrigel introduces high batch-to-batch variability [30,31]. As a mouse-derived material, it contains growth factors that may alter signaling pathways in organoids, influencing cell behavior in response to microenvironmental stimuli and treatments [32,33,34]. To overcome these limitations, we generated GelMA-based bioprinted bladder cancer organoids using two different cell lines that capture the unique characteristics of the tumor across different grades in a human, tunable, and robust 3D environment.

GelMA serves as a powerful alternative to Matrigel, addressing its shortcomings in reproducibility and mechanical tuning. While still derived from a natural source (denatured collagen, gelatin), its manufacturing process is highly standardized, with a more defined chemical composition, resulting in minimal batch-to-batch variation [35]. Unlike Matrigel, GelMA retains collagen peptide sequences such as RGD motifs, which are crucial for cell adhesion, and MMP target sites that enable ECM remodeling. GelMA also avoids background growth factors and exogenous cytokines, while simultaneously enabling the precise loading of these molecules into the 3D construct if so desired for specific cellular support functions [36,37]. Moreover, GelMA allows the tuning of the matrix’s mechanical stiffness through polymer concentration and degree of methacrylation [36,38]. This may provide critical support to maintain organoid integrity and structural stability during experimentation. Some Matrigel-based models reported in the literature described tumor grade-dependent deviations in organoid morphology [29], potentially introducing confounders during evaluation of drug response. In contrast, our system maintains a stable and consistent structural baseline across conditions, allowing for more accurate assessments. In the context of bioprinting, the choice of ECM material and the fast UV crosslinking in BCaOs also help reduce experimental variability.

Notably, the physiological environment provided by GelMA in BCaOs is comparable to that of other matrices. Our group has previously evaluated colorectal tumor organoids fabricated with Hyaluronic Acid-Collagen (HA-Col) blends and observed cell-line-specific behavior and therapeutic sensitivities similar to the GelMA system presented here [39]. For example, HT-29 cells formed spherical groups of cells in areas with higher proliferation, like the clusters observed in our HTB-9 grade 2 organoids. Viability was also cell-line dependent in HA-Col-based organoids, with HCT-116 organoids showing the greatest metabolic activity, consistent with their high aggressiveness. This pattern was mimicked by the more aggressive grade 3 bladder cancer cells within the GelMA-based BCaOs. Despite the similar biological properties between HA-Col and GelMA, HA-Col presents several technical limitations for bioprinting: it exhibits poor printability, relies on slow thermal gelation kinetics, which may increase sample variability, and undergoes significant cell-mediated gel contraction over long culture durations. GelMA retains the critical biological motifs of collagen (such as RGD cell-adhesion and MMP-degradable sequences) while introducing rapid, UV-driven crosslinking. This unique combination maintains physiological relevance while providing precise printability, structural stability, and throughput.

The GelMA concentration in our model was based on previous work in our laboratory and appeared optimal for enabling distinct cell behaviors; however, this feature can be easily adapted to reproduce fibrotic or stiffer environments observed in some bladder cancers across different stages [40]. The protocol can also be adapted to change parameters such as organoid dimensions, volume, and cell density. This is advantageous, particularly when only a small number of cells are available, especially in future iterations when adapted to patient biopsy-derived organoids. Although not performed here, co-culture in our organoid can be easily achieved by suspending cells, such as cancer cells and fibroblasts, together to recreate the tumor environment [40].

Our methodology enables rapid organoid production and scaling. Established organoid systems often require 4–7 days of maturation prior to experimentation [27]. Here, we were able to screen chemotherapies at only 2 days post-generation. Though certain models suffer structural compromise by day 7, our constructs retained optimal morphology for 14 days and remained viable for 21 days. This extended culture window enhances the platform’s utility, making it suitable for studying long-term treatment-exposure dynamics and emerging mechanisms of acquired resistance in vitro.

BCaOs recapitulated the appropriate proliferation and cell-association dynamics of low- and high-grade bladder cancers. Pathologically, low-grade bladder cancers are characterized by more typical organization and differentiation, slower growth, and preserved cell–cell adhesion, whereas high-grade cancers show greater dysplasia, reduced organization, loss of cell–cell adhesion, increased proliferation, and poor differentiation. We observed that grade 2 cells (HTB-9) proliferated in clusters despite being seeded into the organoids as single-cell suspensions, thereby recapitulating the known in vivo cell-association dynamics of lower-grade tumors. These cells also retained a distinct epithelial phenotype, as evidenced by consistent EpCAM membrane staining (Figure 2B). Conversely, grade 3 cells (UM-UC-3) remained single cells while continuing to proliferate. Notably, EpCAM staining in these cells became more diffuse and less membrane-localized (Figure 2A), possibly indicating the loss of the epithelial phenotype and favoring a dysplastic, EMT-prone state characteristic of high-grade tumors. The grade-specific proliferation and cell-association dynamics were maintained throughout the culture, indicating the stability of the organoid system and its reliability for conducting experiments at different disease stages. Thus, BCaOs were effective at reproducing grade-specific bladder cancer behavior in a tunable, human 3D environment.

Bioprinted BcaOs can serve as a platform for drug screening and development. When treated with standard-of-care chemotherapies, BCaOs containing grade 2 cells (HTB-9) showed a significant decrease in viability, whereas grade 3 BCaOs (UM-UC-3) showed a less pronounced response. This is consistent with previous findings in 2D cell culture experiments, in which UM-UC-3 displayed some resistance to cisplatin, gemcitabine [41,42] and other chemotherapies, such as mitomycin-C and docetaxel [41,43,44,45,46]. This supports the validation of the BCaO model, demonstrating that the drug’s therapeutic mechanism is preserved within the biofabricated and more physiologically relevant 3D matrix. Therefore, besides expected drug-response profiles, our model further captures cell organization-related effects that are completely absent in 2D cell cultures, as demonstrated in our histology data. Since the choice of treatment regimen is influenced by tumor depth and grade, our tumor grade-discerning platform enables us to assess the contribution of grade to therapy response, establishing this model as a novel drug-testing tool.

Although novel therapies for cancer are constantly being developed and investigated, few advance to clinical trials or into clinical practice. One of the bottlenecks is the lack of reliable pre-clinical human 3D models that reproduce human tumor response. We explored the use of our BCaOs as a drug development tool by testing the effect of a newly developed drug, LCI139. In our organoids, we observed that grade 2 cells responded to LCI139, whereas the effect was more moderate in grade 3 cells. As higher-grade cells become more dysplastic, they lose epithelial markers and characteristics, which may explain the reduced response in grade 3 cells. Nonetheless, we observed that grade 3 cells responded more to this inhibitor than to the other chemotherapies, suggesting a potentially enhanced tumor-reducing effect of this drug. Staining for CDK9/CDK4/6/PI3K may elucidate the effects of the novel agent LCI139 on these organoids.

Despite their advantages, the GelMA-based BCaOs presented in our study also bear some limitations. First, our model lacks other cells found in the tumor microenvironment of bladder cancers, such as cancer-associated fibroblasts, pericytes, and immune cells [40,47]. All these components have been shown to modify tumor behavior and treatment response [48]. Therefore, future iterations of this model will explore the addition of these cell components. We also noticed GelMA degradation beginning around day 14, significantly impacting organoid usability by day 21. Although this limits the use of the organoids for long-term experimentation, we were able to acquire drug-response data before degradation occurred, indicating that this does not affect the main purpose of this model. Finally, although technically feasible, the present study has not explored the incorporation of patient-derived bladder cancer cells. Follow-up studies should build upon the work here by developing patient-derived BCaOs to aid personalized drug-screening analyses.

In conclusion, bioprinted GelMA-based BCaOs offer a robust and reproducible platform for recapitulating grade-dependent tumor dynamics and treatment responses, establishing them as valuable drug-screening tools for this aggressive and highly diverse cancer. Future iterations of this model will expand its utility by incorporating cancer cells from other primary sites or integrating multicellular components to more faithfully simulate the complex tissue microenvironment.

## 4. Materials and Methods

### 4.1. Cell Culture

Cell lines used were HTB-9 and UM-UC-3 (American Type Culture Collection, Manassas, VA, USA). These are, respectively, grade 2 and grade 3 muscle-invasive bladder cancer cell lines with epithelial morphology. Cells and organoids were cultured in RPMI-1640 media (Gibco; Grand Island, NY, USA) with 10% fetal bovine serum (FBS) and 1% antibiotic/antimycotic (AA, Gibco; Grand Island, NY, USA). Organoids were fabricated once cells reached 70–80% confluence on the initial passage from frozen.

### 4.2. Gelatin Methacrylol (GelMA) Fabrication

GelMA was made in batches in advance and stored for organoid fabrication. GelMA was prepared using gelatin from porcine skin (Sigma-Aldrich; Burlington, MA, USA). Gelatin was dissolved at a concentration of 10% (*w*/*v*) in ultrapure deionized (DI) water at 50 °C until fully dissolved. HCl was then slowly added to adjust the pH of the gelatin solution to 3.5, which is optimal for the methacrylation reaction. For methacrylation of the gelatin, glycidyl methacrylate (GMA) at a volume of 18% of the initial DI water volume was gradually added dropwise overnight. Once all GMA had been added, the methacrylation reaction was allowed to proceed for an additional 12 h. To stop the methacrylation reaction, phosphate-buffered saline (PBS) was added at a volume equivalent to the initial volume of DI water. The solution was placed in dialysis tubing (MWCO 3.4 kDa) and gently stirred in DI water at 37 °C to remove unreacted GMA. Water was changed twice daily, and dialysis was carried out for a total of 10 days. The change in color of the solution from pale white to transparent visually confirmed the removal of unreacted GMA. The remaining solution was filtered while still warm and placed into sterile 50 mL conical tubes. The solution was frozen overnight at −80 °C and subsequently lyophilized for 5 days. Once confirmed that no ice crystals remained, the final GelMA was stored at −20 °C for downstream application. Lastly, a TNBS assay was done to determine the degree of substitution (DS) percentage of 73%.

### 4.3. Crosslinking Method for Organoid Fabrication

**Figure 5:**
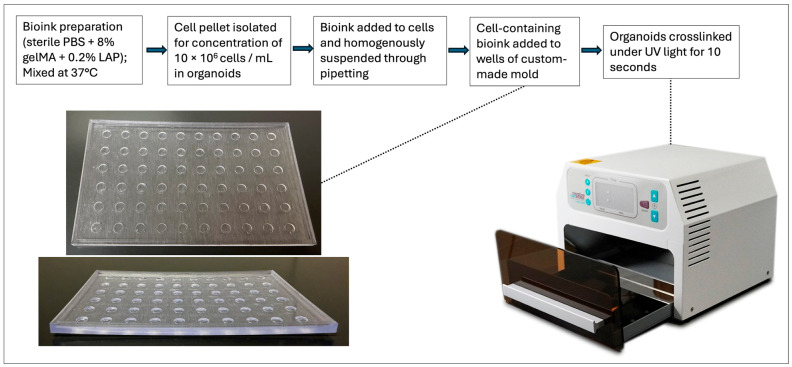
Workflow for organoid generation. Steps for organoid fabrication. Images include a 3D-printed plastic mold and UV box used for crosslinking.

Organoids were fabricated under ultraviolet (UV) light in a mold using a GelMA-based bioink crosslinked with a homogenous cell suspension (Figure 5). Molds were 3D-printed using Biomed clear resin (Form Labs; Somerville, MA, USA). Organoid wells were cylindrical with a diameter of 4 mm and a depth of 0.8 mm, allowing a volume of 10 µL to be used per organoid. To prevent organoid adhesion, the molds were treated with anti-adherence rinsing solution (STEMCELL Technologies; Vancouver, BC, Canada) prior to printing. Sterile PBS was then used to rinse the wells, followed by suctioning and air drying. To prepare our bioink, GelMA at 8% (*w*/*v*) was dissolved in sterile PBS at 37 °C for 30 min. Lithium phenyl-2,4,6-trimethylbenzoylphosphinate (LAP) (Sigma-Aldrich; Burlington, MA, USA) was then added at a concentration of 0.2% (*w*/*v*) and dissolved at 37 °C for 15 min. Cells were lifted, counted, and homogeneously suspended in bioink at a concentration of 10^6^ cells/mL. 10 µL of this final solution was placed into each individual well within the mold, resulting in organoids containing 100,000 cells/organoid. Organoids were exposed to UV light in the UV box for 10 s to crosslink and solidify the bioink into our final organoid product. Immediately after crosslinking, organoids were carefully removed from wells and moved to 48-well non-tissue-culture plates with 500 µL media and maintained in the incubator at 37 °C and 5% CO_2_. Media was RPMI with 10% FBS and 1% AA. Media was replaced daily.

### 4.4. H&E Staining

All histological samples were processed in the same manner to produce Hematoxylin and Eosin (H&E) cross-sectional images. Samples were taken on days 1, 4, 7, 14, and 21. Organoids were fixed in 4% paraformaldehyde overnight at 4 °C. They were then processed and embedded into paraffin wax, where 5 µm cross-sections were placed on slides for staining. Slides were baked at 60 °C for 45 min prior to standard procedure H&E staining. Image acquisition was performed with the E1000 DX Slide Scanner (Epredia; Kalamazoo, MI, USA), and images were processed with the Olympus cellSens Dimension software (v.4.3 Build 31056) (Evident, Tokyo, Japan).

### 4.5. Immunohistochemistry

Paraffin slides were baked at 60 °C for 45 min, after which they underwent deparaffinization and rehydration. Slides underwent antigen retrieval treatment for 2 h with 10 mM sodium citrate (pH = 6.0). PBS containing Triton (0.2%) (PBS-T) was used to permeabilize tissue sections in a humidified chamber for 15 min. Following two 5-min PBS-T washes, slides were incubated for 30 min in protein-blocking solution. Primary antibodies EpCAM anti-rabbit (Abcam; Waltham, MA, USA, cat. no. ab223582) and Ki67 anti-mouse (Abcam, cat. no. ab8191) were diluted 1:200 in antibody diluent (Dako; Carpinteria, CA, USA) and incubated on the sections overnight at 4 °C. The next day, following three 5-min PBS-T washes, the secondary antibodies llama anti-rabbit (Biotium; Fremont, CA, USA cat. no. 20449) and donkey anti-mouse (Biotium, cat. no. 20115) were diluted 1:200 in antibody diluent (Dako; Carpinteria, CA, USA) and incubated on sections for 1 h at room temperature. Following three more 5-min PBS-T washes, sections were stained with DAPI 1 µg/mL (Invitrogen; Eugene, OR, USA) and mounted with DAPI-free Prolong Gold (Invitrogen; Eugene, OR, USA) before adding coverslips. Fluorescent slide images were acquired with the Olympus Slideview VS200 Universal Slide Scanner (Evident, Tokyo, Japan), and images were processed with the cellSens software (Evident, Tokyo, Japan).

### 4.6. Drug Treatment

Drugs included in this study were Gemcitabine (Selleck Chemical; Houston, TX, USA, cat. no. S1714), Cisplatin (Selleck Chemical, cat. no. S1166), Doxorubicin (Selleck Chemical; Houston, TX, USA, cat. no. S1208), and LCI 139. Treatments were applied 48 h after organoid fabrication, and viability was assessed 72 h after treatment. For each of the two cell lines used in the study, 5 organoids were used per drug concentration. Treatment took place in a non-treated 48-well plate with 500 µL of media used per organoid. Gemcitabine from 10 mM stock solution was diluted with sterile ultrapure water to concentrations of 1, 5, and 10 µM. Cisplatin powder was first dissolved to create 10 mM stock solution. Then, this stock solution was diluted with sterile ultrapure water to concentrations of 1, 5, and 10 µM. Doxorubicin from 10 mM stock solution was diluted with dimethyl sulfoxide (DMSO) to concentrations of 0.5, 1, and 10 µM. LCI 139 from 10 mM stock solution was diluted with dimethyl sulfoxide (DMSO) to concentrations of 1 and 5 µM.

### 4.7. Viability ASSAY

Following 72 h of drug treatment, the drug-containing media was fully replaced with standard culture media containing 10% Alamar Blue assay. After 8 h, 200 µL of the supernatant was removed, and the fluorescence (560 nm excitation, 590 nm emission) was read on the SpectraMax MiniMax 300 Imaging Cytometer plate reader (Molecular Devices; San Jose, CA USA) to assess cell viability.

### 4.8. Statistical Analysis

Sample size (*n*) for statistical analysis is included in the associated figure legends. The significance of pairwise data comparisons was evaluated using a two-tailed Student’s *t*-Test. All data are presented as mean ± standard deviation. Further details of statistical analysis and significance values are described in the figure legends.

## Data Availability

The original contributions presented in this study are included in the article. Further inquiries can be directed to the corresponding author.
